# Extended Curettage and Fibular Grafting in Enchondroma of the Acromion

**DOI:** 10.7759/cureus.7630

**Published:** 2020-04-11

**Authors:** Kuldeep Bansal, Pratyush Shahi, Anil K Jain, Ish K Dhammi, Saurabh Kumar

**Affiliations:** 1 Orthopaedics, University College of Medicine Science and Guru Teg Bahadur Hospital, Delhi, IND

**Keywords:** enchondroma, acromion, extended curettage, fibular grafting

## Abstract

We report the case of a six-year-old male child with progressive pain and swelling of the right shoulder for six months. On examination, there was a 7x7 cm globular, tender swelling with firm consistency over the posterolateral corner of the right shoulder. Radiographs showed an expansile lytic lesion in the acromion process of the scapula. Biopsy showed lobules of hypocellular cartilage separated by fibroconnective stroma suggestive of an enchondroma. Extended curettage and fibular bone grafting of the lesion was done. At one-year follow-up, the patient was symptom-free and had full, painless shoulder range of motion. To the best of our knowledge, there is no published record of an enchondroma of the acromion.

## Introduction

Enchondroma is a benign cartilaginous lesion commonly found in small bones of the hands and feet [1]. It is common and affects all age groups. Generally being asymptomatic, it is discovered incidentally during an unrelated radiographic examination or in case of a pathological fracture. Proximal locations such as proximal humerus and scapula are extremely rare sites [2]. Radiographs show a lytic lesion with intralesional calcification. Asymptomatic lesions can be managed conservatively with follow-up and serial radiographs. If the lesion grows or if it becomes symptomatic, extended curettage usually is curative [3]. We report the case of a six-year-old male child with enchondroma of the acromion, which, to the best of our knowledge, has never been published.

## Case presentation

A six-year-old male child presented to our orthopaedics department with the complaints of pain and swelling over the right shoulder for six months. Both pain and swelling were of insidious onset and had gradually progressed. Pain was of dull-aching type. This was not associated with radiation of the pain or any history of trauma, fever or night pains. On clinical examination, there was a globular, tender swelling, about 7x7 cm in size with firm consistency, present over the posterolateral corner of the right shoulder. Overlying skin was normal and there were no dilated veins, scar marks or sinuses. There was no local rise in temperature, lymphadenopathy, distal neurovascular deficit or any other swelling elsewhere in the body.

Blood investigations showed normal counts and kidney and liver functions. Serum calcium, erythrocyte sedimentation rate, C-reactive protein, alkaline phosphatase and parathyroid hormone were normal.

**Figure 1:**
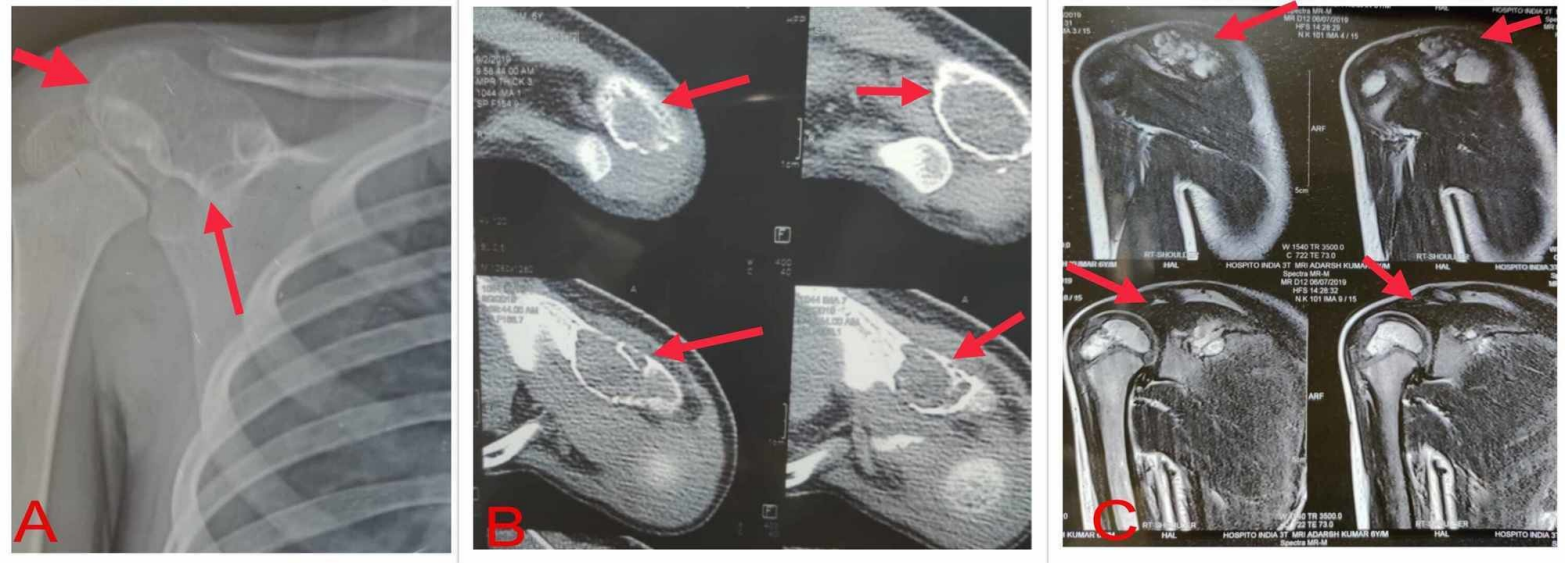
Preoperative radiographic assessment (A) X-ray showing an expansile lytic lesion in the acromion; (B) CT scan showing cortical breach and incomplete septations; (C) MRI showing the lesion to be hypointense on T1 and hyperintense on T2.

X-ray of the right shoulder showed an expansile lytic lesion in the acromion process of scapula with no periosteal reaction or soft tissue component. CT scan showed an expansile lytic lesion with incomplete septations and a cortical breach. MRI showed the lesion to be hypointense in T1 images and hyperintense in T2 images (Figure 1). A skeletal survey did not reveal any other lesion.

The differential diagnoses of simple bone cyst, aneurysmal bone cyst and chondroblastoma were kept in mind. A core biopsy from the posterolateral corner of the shoulder was done. Straw coloured fluid within the bony cavity was found. Biopsy specimen was sent for histopathological examination, which revealed lobules of hypocellular cartilage separated by fibroconnective stroma, features suggestive of enchondroma.

**Figure 2:**
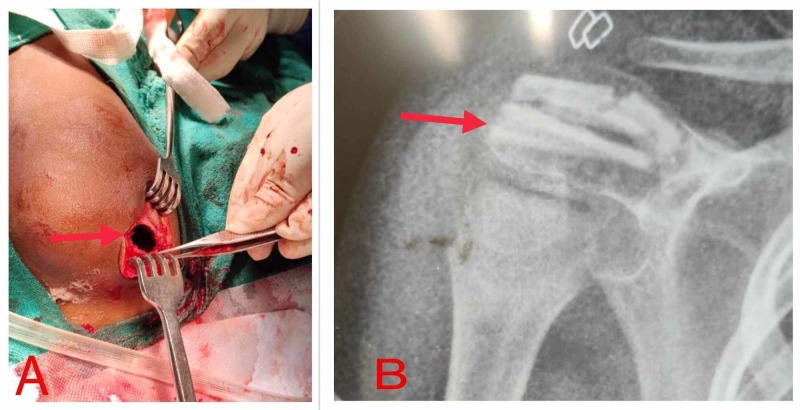
Extended curettage and fibular grafting (A) Posterolateral surgical approach; (B) postoperative X-ray.

Due to persistent pain in the subsequent follow-ups, an extended curettage with hydrogen peroxide and fibular bone grafting of the lesion was done (Figure 2). The patient was discharged on the third day after wound inspection. Sutures were removed on day 14, and passive range of motion exercises of the shoulder were started as tolerated by the patient.

**Figure 3:**
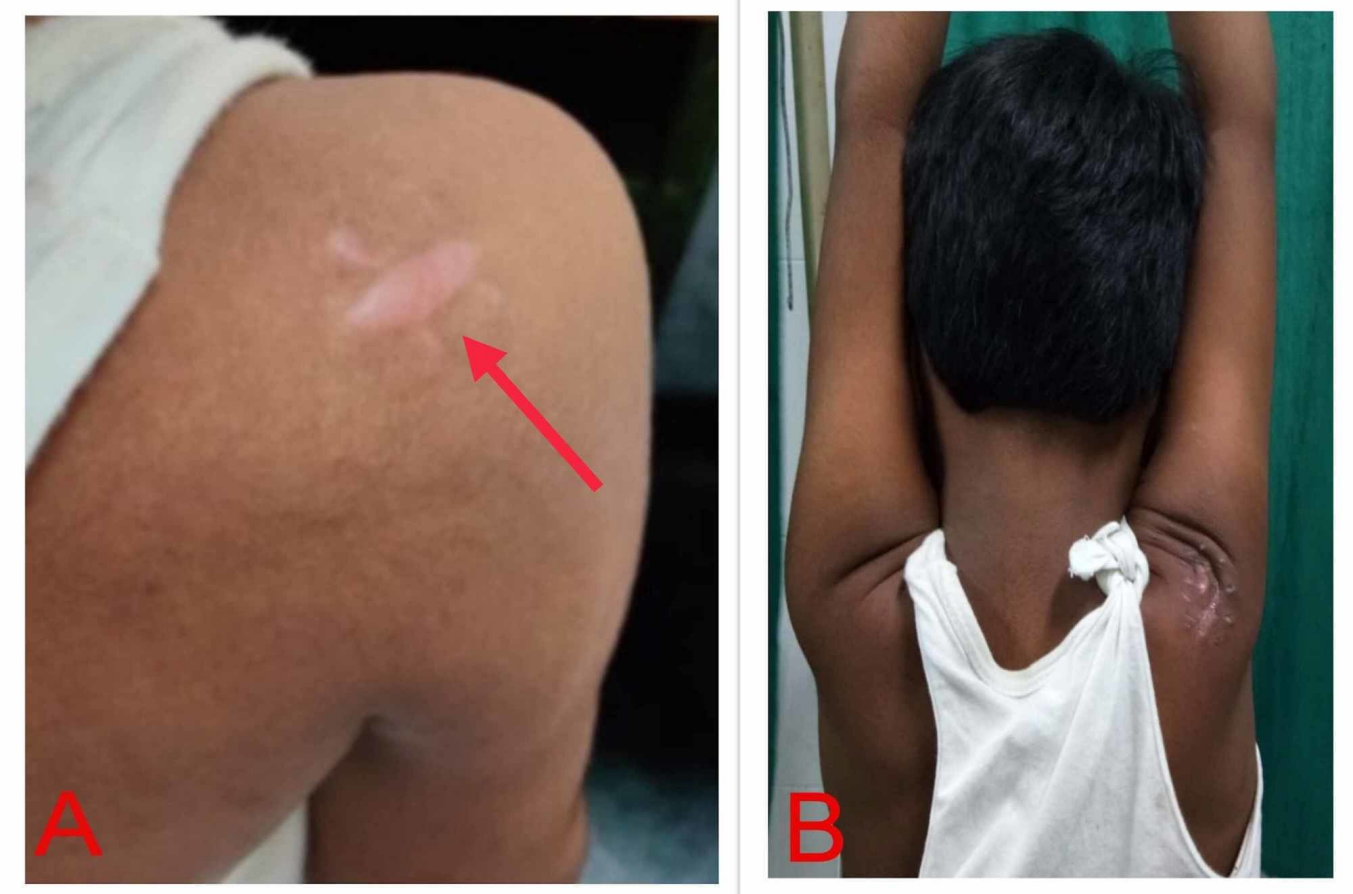
Clinical assessment at one-year follow-up (A) Well-healed surgical scar; (B) full elevation at the right shoulder.

At one-year follow-up, the patient remained symptom-free, having complete and painless range of motion of the shoulder joint (Figure 3). The patient was explained about the need of a long-term regular follow-up. 

## Discussion

Chondroma is a benign cartilaginous lesion, and is called an enchondroma when arising from the medullary canal. It is called a perisosteal or juxtacortical chondroma when it arises from the surface of the bone [4]. Enchondroma is a benign tumour of the bone containing hyaline cartilage. Multiple enchondromatosis is known as Ollier’s disease, and when associated with hemangioma of overlying soft tissues, it referred to as Maffuci’s syndrome [5]. It is the most common tumour of the hands and feet. Proximal femur, pelvis, proximal humerus and scapula are rare sites. Proximal sites are more prone to recurrence and malignant change (chondrosarcoma), and hence should be monitored regularly [6].

Various lesions arising from the acromion, such as osteochondroma, simple bone cyst (SBC), aneurysmal bone cyst (ABC), giant cell tumour with secondary ABC, chondroblastoma, metastasis and rarely multiple myeloma, have been described in the literature. Our six-year-old patient with expansile lytic lesion of the acromion had differential diagnoses of SBC, ABC, and chondroblastoma. Both SBCs and ABCs present as lytic lesions, but ABCs are blood-filled cavities with septations and fluid-fluid levels on MRI scan [7]. Chondroblastoma is a rare, benign tumour with predilection for proximal humerus, but has also been reported in the acromion [8].

Our case was proved to be a solitary enchondroma on biopsy. It could be managed conservatively with serial radiographs, but an extended curettage and fibular bone grafting was done due to persistent pain and its proximal location which made it prone to a malignant change. Various other options of treatment for an acromial tumour such as en bloc excision with or without stabilisation have been described [9].

## Conclusions

Enchondroma represents a rare differential diagnosis of a lytic lesion of the acromion and must be kept in mind. Extended curettage and bone grafting in a symptomatic patient yields excellent clinical outcomes. A long-term and regular follow-up is required to monitor for recurrence or malignant transformation.
